# Supplementary material for: Retinoid-X receptor agonists increase thyroid hormone competence in lower jaw remodeling of pre-metamorphic Xenopus laevis tadpoles
Source: PLoS One. 2022 Apr 13;17(4):e0266946. doi: 10.1371/journal.pone.0266946 (PMC9007347; doi:10.1371/journal.pone.0266946)
Supplement: Attachment — Submitted filename: Response to Reviewers.docx [file pone.0266946.s002.docx]

March 9, 2022

**Brenda Mengeling**

Associate Project Scientist

Dear Dr. Klymkowsky,

Thank you for allowing us to revise our manuscript for PLOS ONE:

**Title: Retinoid-X receptor agonists increase thyroid hormone competence in lower jaw remodeling of pre-metamorphic *Xenopus laevis* tadpoles**

**Authors:** Brenda J. Mengeling (corresponding author), Lara F. Vetter, and J. David Furlow

We found the reviewers’ responses helpful, and we have now included revised analysis (Reviewer 1, point 1) and new data (Reviewer 2). The new data, Alcian blue staining of cranial cartilages in the tadpole, became Figure 2, so all the figure numbers of the original figure 2 onward have advanced by one number. We were able to solicit the generous assistance of our colleagues here at Davis in finding the most suitable microscope/color camera we needed, and thus the staining turned out quite well and dramatically shows the remodeling of the lower jaw cartilages and the resorption of the branchial arches.

Responses to Reviewer 1:

1. Normalization to the size of the cartilage area counted was a very good idea for our proliferation data (previously Figure 2, now Figure 3). We have done this, and our revised results now show significant inhibition of T3-induced proliferation by the RXR antagonist UVI.
2. We have provided an explanation of the differences between the endogenous *thibz* gene promoter and the promoter in the TRE-Luc reporter in both the results (lines 462-466) and the discussion (lines 576-590). We also explain in the discussion our rationale for using the Luc reporter as it performs as an excellent surrogate to general gene expression changes with treatment (i.e., potentiation or inhibition of T3-action by RXR agonists or antagonist, respectively), and its activity follows morphological changes associated with the treatments.
3. We discuss in the results (lines 363-370) the discrepancy between non-significant changes in aurkb message expression and significant changes in Aurkb kinase activity. The simplest explanation is that Aurkb kinase activity isn’t directly tied to its message level at the level of change we see in the kinase activity.
4. We corrected all typos that we found in the text.

Response to Reviewer 2:

We have added a figure (current Figure 2) of Alcian blue cartilage staining of the tadpole head from the ventral side so the reader can visualize the cranial cartilages and how they change in response to T3 and the RXR agonists (lines 219-228, 316-341, 534-536). We measured the length of the Meckel’s cartilage, and we show that its length does not change with treatment. We also validated our LJ angle measurement using dorsal head photos of fixed tadpoles (Figure 1) by measuring the angle formed by the Meckel’s and infrarostral cartilages, showing that both sets of measurement agree with each other.

In the introduction (lines 156-167) we briefly explain the timing of the roles of retinoic acids and THs in cranial development, specifying that one advantage of assaying at 1-week post-fertilization is it’s a time when the jaw cartilages are already formed from the action of retinoic acids, but that T3 action of cartilage maturation and bone ossification has not occurred. Therefore, we are perturbing the system at a “quiet” time of isometric growth and not morphological change in the jaw. We also better explain the non-natural changes T3 induces at this stage, and how we are still able to use it as a readout of T3 action and disruption. We did not discuss the specification and migration of the neural crest, because in our system that has already occurred before we start treatments, and therefore, cannot be perturbed by such. To keep the introduction at a reasonable length, we shortened it in other paragraphs, as the tracking shows.

We hope these responses meet with the reviewers’ approval, and that we can gain acceptance for publication in PLOS ONE.

Sincerely,

Brenda Mengeling
